# *QuickStats:* Percentage* of Children and Adolescents Aged ≤17 Years Who Visited an Urgent Care Center or a Clinic in a Drug Store or Grocery Store in the Past 12 Months,^†^ by Age Group and Year — National Health Interview Survey,^§^ United States, 2021–2022

**DOI:** 10.15585/mmwr.mm7303a5

**Published:** 2024-01-25

**Figure:**
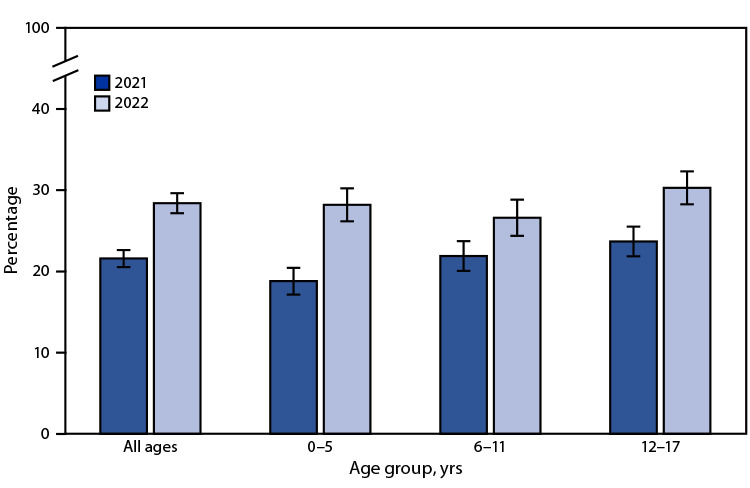
The percentage of children and adolescents aged ≤17 years who had at least one visit to an urgent care center or a clinic in a drug store or grocery store in the past 12 months increased from 21.6% in 2021 to 28.4% in 2022. This increase was noted for all age groups during 2021–2022. In 2021, urgent care or retail health clinic visits were lower among children aged 0–5 years than those aged 6–11 years and 12–17 years. In 2022, visits for those aged 12–17 years (30.3%) were higher than for those aged 6–11 years (26.6%). Other observed differences among age groups were not significant.

**Source:** National Center for Health Statistics, National Health Interview Survey, 2021–2022 data. https://www.cdc.gov/nchs/nhis/index.htm

